# Integrated social-ecological data for regional natural resource management

**DOI:** 10.1016/j.dib.2023.109806

**Published:** 2023-11-11

**Authors:** Vanessa M. Adams, Stuart Allen, Ruth Steel, Natalie Stoeckl, Silva Larson

**Affiliations:** a School of Geography, Planning, and Spatial Sciences, University of Tasmania, Private Bag 51, Hobart, TAS 7001, Australia; b College of Business and Economics, University of Tasmania, Private Bag 51, Hobart, TAS 7001, Australia

**Keywords:** Australia, Biodiversity, Economics, Households, Integrated regional datasets, Regional planning, Environmental management

## Abstract

Natural resource managers need information about both human and natural systems and interactions between those systems. Much data is available, but mostly from disparate sources and data have often been collected at different time steps and at different geographic scales. We used insights from the literature to select 270 relevant variables, available at national scale, from 33 unique (Australian) data sources. There were numerous with repeat measures, so in total we have 425 variables: 143 specific to 2016, 148 specific to 2021, and 134 available for both periods. We used GIS to summarize the variables spatially based on two geographic boundaries: one describes 63 Natural Resource Management Regions; the other describes 419 (sub) bioregions (formally, IBRA – Interim Biogeographic Regionalisation for Australia). Data deficiencies prevented us from being able to report on all variables for all regions. In the NRM dataset many regions are offshore islands, about which data are not generally available. Moreover, many IBRA regions are small and household level data are not always available at that scale. For analyses requiring a complete dataset at a single time step, our 2021 dataset for NRM regions includes 270 unique variables that describe 56 regions. Our IBRA data includes 214 variables describing 409 regions. To help managers select appropriate data for specific problems/contexts, the metadata file also categorises variables according to (a) whether they pertain to the social or ecological system, or interactions; (b) the segment of society described (where relevant); and (c) the frequency with which data are updated.

Specifications Table

**Table:** 

Subject	Nature and Landscape Conservation
Specific subject area	Integrated regional databases to support investment decisions in Nature and Landscape Conservation
Type of data	Our data pack includes 6 unique files: • Metadata.xlsx: provides Data citations and license details for 37 unique data sources and all variable details as processed in this dataset • data_description_nrm_2023.html: Data description of variables and summary methods as output from summary code • ibra_compile_2023.csv: tabular summary for IBRA regions • nrm_compile_2023.csv: tabular summary for NRM regions • NESP RL Hub IBRA subregions 2023.lpk: spatial layer package for IBRA regions • NESP RL Hub NRM regions 2023.lpk: spatial layer package for NRM regions
How the data were acquired	We downloaded public datasets, in various formats, through on-line portals: Australian Bureau of Statistics (ABS), Australian Electoral Commission, Australian Institute of Health and Welfare, Australian Curriculum, Assessment and Reporting Authority, Bureau of Meteorology, CSIRO, Department of Agriculture, Fisheries and Forestry, Department of Climate Change, Energy, the Environment and Water, Geoscience Australia, GrantConnect, National Native Title Tribunal, Dryad, Natural Hazards Research Australia and Research Data Australia). HILDA data were sourced under licence. We used datasets released from 2015 to 2022, and then analyzed them to compile them in spatially consistent ways for two regional units (Natural Resource Management boundaries and IBRA subregion boundaries).
Data format	Analyzed
Description of data collection	A compilation of Australian data that are (a) publicly available; and (b) use consistent measures across the continent. The compilation is deliberately broad - aiming to include variables that describe each of four 'capitals' (natural, human, social / institutional and financial/built). Our data set includes 6 unique files: 1) Metadata.xlsx: provides Data citations and license details for 37 unique data sources and all variable details as processed in this dataset 2) data_description_nrm_2023.html: Data description of variables and summary methods as output summary code 3) ibra_compile_2023.csv: tabular summary for IBRA regions 4) nrm_compile_2023.csv: tabular summary for NRM regions 5) NESP RL Hub IBRA subregions 2023.lpk: spatial layer package for IBRA regions 6) NESP RL Hub NRM regions 2023.lpk: spatial layer package for NRM regions.
Data source location	• Institution: University of Tasmania • City/Town/Region: Hobart, Tasmania • Country: Australia
	The following raw data sources were used (dataset title and URL provided):
	1. 1270.0.55.005 - Australian Statistical Geography Standard (ASGS): Volume 5 - Remoteness Structure, July 2016 (https://www.abs.gov.au/AUSSTATS/abs@.nsf/DetailsPage/1270.0.55.005July%202016?OpenDocument) 2. General Community Profile for Statistical Area 1 (SA1) 2016 and 2021 census datapacks (https://datapacks.censusdata.abs.gov.au/datapacks) 3. Federal Election House of Representatives Members Elected 2016 and 2019 elections data (https://results.aec.gov.au/24310/Website/HouseDownloadsMenu-24310-Csv.htm) 4. Federal Election Senate First Preferences By Division By Candidate By Vote Type 2016 and 2019 elections data (https://results.aec.gov.au/24310/Website/SenateDownloadsMenu-24310-Csv.htm) 5. 9 arcsecond gridded HCAS 2.1 (2001–2018) base model estimation of habitat condition for terrestrial biodiversity, 18-year trend and 2010–2015 epoch change for continental Australia. v7. (https://doi.org/10.25919/nkjf-f088) 6. Australia - Present Major Vegetation Groups - NVIS Version 6.0 (Albers 100 m analysis product) and NVIS version 4.2 (Albers 100 m analysis product) (https://www.environment.gov.au/fed/catalog/search/resource/downloadData.page?uuid=%7B991C36C0-3FEA-4469-8C30-BB56CC2C7772%7D) 7. Australia - Species and Communities of National Environmental Significance - Downloadable Grids - Number occurring across Australia (https://www.environment.gov.au/fed/catalog/search/resource/details.page?uuid=%7B58C0EB42-2B34-4F12-9869-AEB140348E57%7D) 8. Australia's Indigenous land and forest estate (2020) (https://doi.org/10.25814/cfm3-db86) 9. Australian Critical Minerals Operating Mines And Deposits (http://pid.geoscience.gov.au/dataset/ga/144485) 10. National Indicative Aggregated Fire Extent Datasets (2020) https://www.environment.gov.au/fed/catalog/search/resource/details.page?uuid=%7B9ACDCB09-0364-4FE8-9459-2A56C792C743%7D 11. Australian fire history 1968–2019 (areas burned in the last 5, 15 or 50 years) (https://onlinelibrary.wiley.com/doi/10.1111/ddi.13265) 12. Australian Groundwater Explorer (http://www.bom.gov.au/water/groundwater/explorer/map.shtml) 13. Australian Hydrological Geospatial Fabric (Geofabric) v3.2 (http://www.bom.gov.au/water/geofabric) 14. Catchment Scale Land Use of Australia (https://doi.org/10.25814/aqjw-rq15) 15. Climatologies at high resolution for the earth's land surface areas (CHELSA v2.1) (https://envicloud.wsl.ch/#/?prefix=chelsa%2Fchelsa_V2%2FGLOBAL%2F) 16. Collaborative Australian Protected Areas Database (CAPAD) 2020 - Terrestrial (https://www.environment.gov.au/fed/catalog/search/resource/details.page?uuid=%7B4448CACD-9DA8-43D1-A48F-48149FD5FCFD%7D) 17. Commonwealth Heritage List Spatial Database (CHL) - public (https://www.environment.gov.au/fed/catalog/search/resource/details.page?uuid=%7B92C7656F-7302-4763-B700-EE59B18BED2C%7D) 18. DEA Waterbodies (Landsat) 2.0.0 (https://cmi.ga.gov.au/data-products/dea/693/dea-waterbodies-landsat) 19. Disaster Resilience Index (https://adri.bnhcrc.com.au/#!/maps) 20. GrantConnect, Grant Award Published, Department of Climate Change, Energy, the Environment and Water, 2017–22 (https://www.grants.gov.au/ga/list) 21. Hospital resources 2020–21 tables (https://www.aihw.gov.au/reports-data/myhospitals/content/data-downloads) 22. Land tenure of Australia 2010–11 to 2015–16, 250 m (https://www.awe.gov.au/abares/aclump/land_tenure_2010-11_2015-16) 23. Mapped Agricultural Land Capability Classification Data for Australia V.20 (https://dx.doi.org/10.25959/kjdc-k508) 24. Modelled plant distributions for Australian flora (https://doi.org/10.1111/jvs.13018)
	25. National Heritage List Spatial Database (NHL) - public (https://www.environment.gov.au/fed/catalog/search/resourcedetails.page?uuid=%7BDBB2344C-D0BE-4927-B0C5-44F9F8E1183F%7D) 26. Native Title Determination Outcomes (http://www.nntt.gov.au/assistance/Geospatial/Pages/DataDownload.aspx) 27. Natural Resource Management (NRM) Regions (2020) (https://www.environment.gov.au/fed/catalog/search/resource/downloadData.page?uuid=%7BAB80DA43-CB00-455D-8A3C-70162EB8D964%7D) 28. Interim Biogeographic Regionalisation for Australia (IBRA), Version 7 (Regions) (https://www.environment.gov.au/fed/catalog/search/resource/details.page?uuid=%7B4A2321F0-DD57-454E-BE34-6FD4BDE64703%7D) 29. School Location 2021 (xlsx, 2 MB) List of all Australian schools, Long/Lat, LGA (https://www.acara.edu.au/contact-us/acara-data-access) 30. Airports of Australia 2021 (Xlsx) List of all Australian airports, Long/Lat (http://www.fallingrain.com/world/AS/airports.html) 31. This dataset uses unit record data from The Household, Income and Labour Dynamics in Australia (HILDA) Survey, RESTRICTED RELEASE 20 (Waves 1–20) (https://dataverse.ada.edu.au/dataset.xhtml?persistentId=doi%3A10.26193%2FPI5LPJ) conducted by the Australian Government Department of Social Services (DSS). The findings and views reported in this spatial dataset, however, are those of the authors and should not be attributed to the Australian Government, DSS, or any of DSS’ contractors or partners. DOI: 10.26193/YP7MNU, ADA Dataverse. Pursuant to the license terms for the restricted release data we have only summarized to NRM regions. IBRA sub-regions are at a scale smaller than postcode for some states and thus cannot be summarized or reported as the dataset is not representative at this level. 32. Threatening processes to taxa of conservation concern in Northern Australia (https://doi.org/10.25903/5b72631b2dd70) 33. Value of assets protected by biosecurity (https://www.sciencedirect.com/science/article/pii/S2212041623000013?via%3Dihub)
Data accessibility	Repository name: University of Tasmania Research Data Portal Data identification number: DOI 10.25959/yeng-y344 Direct URL to data: https://dx.doi.org/10.25959/yeng-y344

## 1 Value of the Data

• Funds allocated to research, planning and on-ground environmental action must be spent efficiently to achieve the best outcomes for ecosystems and biodiversity. Our dataset provides critically important contextual information to help target ‘efficient’ environmental planning and investment.
• Our datasets are most able to inform Australian natural resource managers who make decisions at relatively large geographic scale (regional, state, national).
• Our datasets allow regions in Australia to be described using consistent and comparable metrics, so can be used to identify areas that may need specialized on-ground interventions (e.g., restoration, conservation, particular types of planning to negotiate competing interests).
• Our datasets can also be used to identify regions that share similar social and/or ecological characteristics.

## 2 Objective

Our objective was to create integrated datasets to inform and support those interested in promoting biodiversity and in managing natural resources across Australia. There is no shortage of potentially relevant data; but much has been collected by different agencies, at different geographic scales, at different points, and for different purposes. We sought to value-add to those datasets by integrating and further classifying to support broad usage. Our overarching objective, can thus be broken down into three sub-objectives, namely to:

1) systematically identify and select variables relevant to natural resource managers;
2) use those variables to develop indicators, consistently measured across the Australian continent; and
3) integrate indicators within two regional datasets to support decision making.

## 3 Data Description

The datasets are available at:

Within the data record there are 6 files:

• Metadata.xlsx: provides data citations and license details for 37 unique data sources, and all variable details (425 in total) as processed in this dataset.
• data_description_nrm_2023.html: Data description of variables and summary methods as output from summary code
• ibra_compile_2023.csv: tabular summary for 419 IBRA regions with 323 variables
• nrm_compile_2023.csv: tabular summary for 63 NRM regions with 424 variables
• NESP RL Hub IBRA subregions 2023.lpk: spatial layer package for IBRA regions
• NESP RL Hub NRM regions 2023.lpk: spatial layer package for NRM regions

Users may choose to interact with the data through the metadata table to identify and choose sources of data for their own purposes. Table 1 below lists all datasets summarized in our compilation, providing the link, licensing agreements (where applicable), and count of variable by 2016, 2021, or both. These summaries are available on the summary tab of the metadata.xlsx file as well.

**Table 1:** Data summary of the 33 data sources (and where multiple versions were downloaded, we provide reference for each). We indicate the type of data license that the data is available under and that we have adhered to in our analysis and reproduction as regional summary statistics. Counts of variables available by time step are provided as 2016, 2021, Both, and Total.

Data reference	Data license	2016	2021	Both	Total
Natural Resource Management (NRM) Regions (2020) [2]	CC BY 4.0			1	1
Interim Biogeographic Regionalisation for Australia (IBRA), v. 7 [3]	CC BY 3.0			1	1
Australian Bureau of Statistics (ABS) census, [1] and [4]	CC BY 4.0	41	40		81
Australian Statistical Geography Standard (ASGS): Volume 5 - Remoteness Structure 2016 [5]	CC BY 2.5 AU			6	6
Catchment Scale Land Use of Australia – update 2020 [6]	CC BY 4.0			41	41
Climatologies at high resolution for the earth's land surface areas (CHELSA v2.1) [7]	CC BY 4.0			9	9
National Heritage List Spatial Database (NHL) - public [8]	CC BY 3.0 AU			1	1
Commonwealth Heritage List Spatial Database (CHL) – public [9]	CC BY 3.0 AU			1	1
Australian Critical Minerals Operating Mines And Deposits [10]	CC BY 4.0			4	4
Land tenure of Australia 2010–11 to 2015–16 [11]	CC BY 4.0			4	4
Mapped Agricultural Land Capability Classification Data for Australia V.20 [12]	CC BY 4.0			2	2
National Vegetation Information System v 6.0 [13] and v. 4.2 [14]	CC BY 3.0 AU	32	32		64
Modelled plant distributions for the Australian flora [15]	By permission			1	1
Australia - Species and Communities of National Environmental Significance - Downloadable Grids - Number occurring across Australia [16]	CC BY 3.0			4	4
Collaborative Australian Protected Areas Database (CAPAD) 2020 - Terrestrial [17]	CC BY 4.0 AU			1	1
Australian Hydrological Geospatial Fabric (Geofabric) v3.2 [18]	CC BY 4.0			1	1
National Indicative Aggregated Fire Extent Datasets [19]	CC BY Attribution			1	1
Australian fire history 1968–2019 (areas burned last 5, 15, 50 years) [20]	By permission			3	3
Airports of Australia [21]	Public			5	5
Value of assets protected by biosecurity [22]	CC BY 4.0 AU			12	12
Australian Electoral Commission House elections 2016 [23] and 2019 [24]	CC BY 4.0	8	8		16
Australian Electoral Commission Senate elections 2016 [25] and 2019 [26]	CC BY 4.0	12	18		30
Native Title Determination Outcomes [27]	CC BY 4.0			4	4
Australia's Indigenous land and forest estate (2020) [28]	CC BY 4.0			14	14
Disaster Resilience Index [29]	CC BY NC 4.0			3	3
Threatening processes to taxa of conservation concern in Northern Australia [30]	CC BY 4.0 AU			2	2
9 arcsecond gridded HCAS 2.1 (2001–2018) v7 [31]	CC BY 4.0			2	2
Grant Connect, Grant Award Published, Department of Climate Change, Energy, the Environment and Water, 2017–22 [32]	CC BY 3.0 AU			2	2
School Location 2021 List of all Australian schools, Long/Lat, LGA [33]	CC BY 4.0			5	5
Hospital resources 2020–21 tables [34]	CC BY 4.0			1	1
Australian Groundwater Explorer [35]	CC BY 4.0			1	1
DEA Waterbodies (Landsat) 2.0.0 [36]	CC BY 4.0			1	1
This dataset uses unit record data from The Household, Income and Labour Dynamics in Australia (HILDA) Survey, RESTRICTED RELEASE 20 (Waves 1–20) conducted by the Australian Government Department of Social Services (DSS) [37]. The findings and views reported in this spatial dataset, however, are those of the authors and should not be attributed to the Australian Government, DSS, or any of DSS’ contractors or partners. DOI: 10.26193/YP7MNU, ADA Dataverse. Pursuant to the license terms for the restricted release data we have only summarized to NRM regions. IBRA sub-regions are at a scale smaller than postcode for some states and thus cannot be summarised or reported as the dataset is not representative at this level.	Licenced	50	50		100

**Grand Total**		**143**	**148**	**134**	**425**

**Fig. 1:**
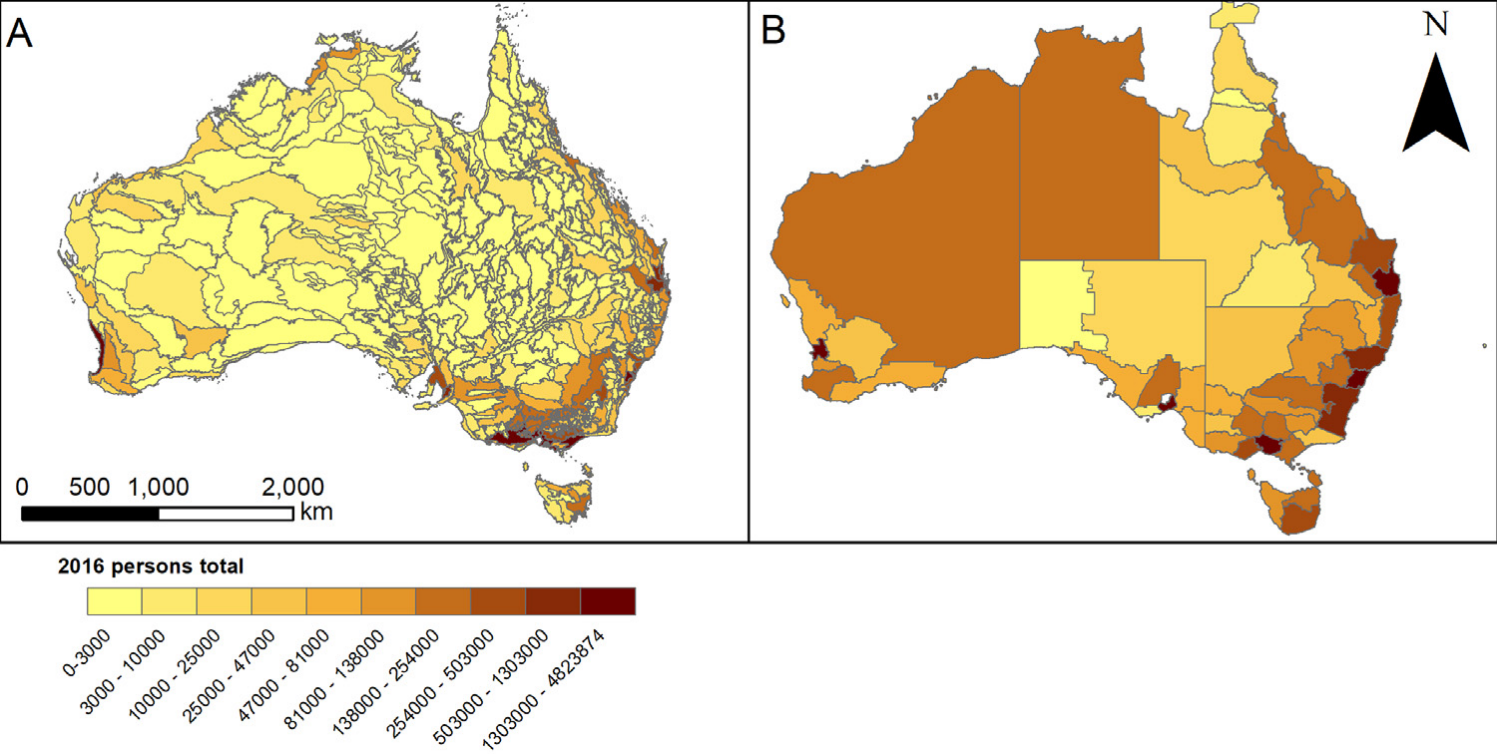
Regional summary data as viewed when.lpk files opened. A) 2016 total persons summarised at IBRA regions from census data [1], B) 2016 total persons summarised at NRM regions from census data [1].

Users may interact with the data in csv format for statistical analyses, or they can visualize spatial patterns using our.lpk files (Fig. 1– displays for 2016 persons total summaries as opening view for.lpk files).

## 4 Experimental Design, Materials and Methods

We first used insights from the NRM literature to develop a comprehensive framework (a *wish list*) to guide the identification and selection of relevant variables [38]. Guided by our ‘wish list’, we searched for relevant Australian datasets available at national scale. We found more than 60 products that had been collected by more than 40 agencies, at different geographic scales and at different points in time. We used the *wish list t*o guide the selection of variables from those data products, collating data for different periods (2016 and 2021), where available [39]. This resulted in a final set of 37 unique data sets from 33 data providers (4 have versions available at two time points). From these unique data sets we compiled 425 variables. Our data included two time-steps for 6 data sets: census data, households, lower and upper house elections, vegetation, habitat condition. Taking into account variables available for each time step (2016 and 2021) and those shared for each time step we have: 143 specific to 2016, 148 specific to 2021, and 134 shared across time steps (Table 1).

**Fig. 2:**
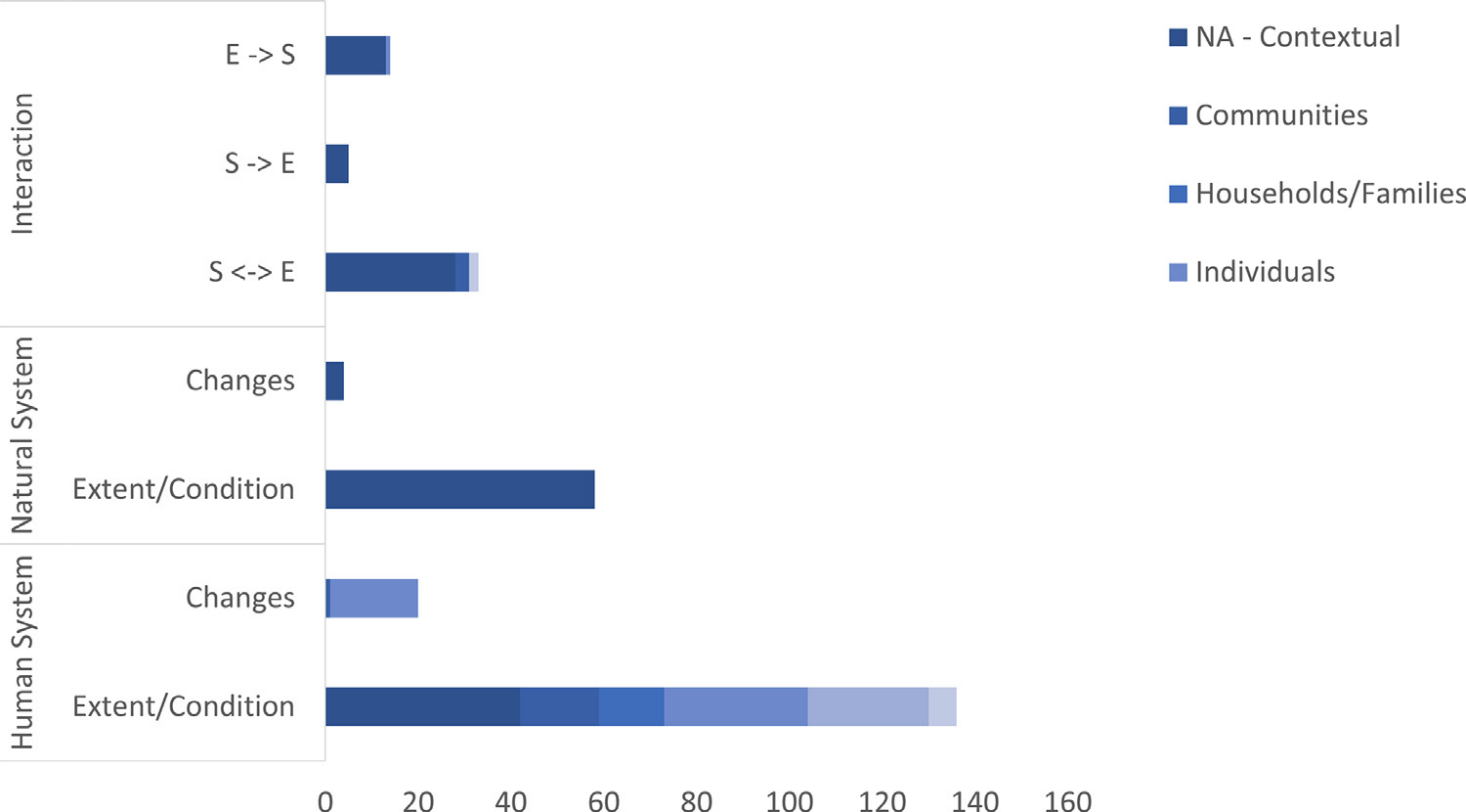
Number of variables describing various parts of the social-ecological system (human, natural or interaction) and segment of society that is described (where applicable). Adapted from Stoeckl, Adams, Larson, Allen, Jia, Boothroyd and Steel [39] with permission.

We sought to determine if there was sufficient breadth and depth within the dataset to adequately characterize regions – assuming that the primary aim is to provide information to support natural resource managers. Research on complex social-ecological systems, highlights the need to describe the social (human) system, the ecological (natural) system, and interactions between the two systems, including descriptions of institutional/governance arrangements relevant to natural resources [39]. We thus categorised variables according to the part of the system they relate (social, ecological, interaction) – see the column titled *Broad Classification* in our metadata file. For variables describing either the social or ecological systems, we also considered whether the variables were describing the extent/condition of the system or describing changes in the system. When categorizing ‘interactions’, we distinguished between variables thought to describe what humans/society do to or for the environment (*S → E*), what the environment does to or for society (*E → S*), or whether they described two-way interactions, *S ↔ E* (e.g., land uses – where people ‘do’ things to land, which provides things for people). This additional information (change/extent versus change; *S → E, E → S, S ↔ E)* is recorded in the metadata file in a column titled *Description of system or interaction.* Fig. 2 shows the breakdown of our variables in the 2021 dataset as categorised.

Research relating to social-ecological systems also highlights the critical role of ‘actors’ [38]. Where applicable, variables are also categorised in this way – the relevant column in the metadata file is titled: *Segment of Society Described*. The categories we considered were communities, households/families, individuals, political representatives, or workforce. Where it was not relevant to categorize a segment of society for a variable, we entered NA for not applicable. Finally, we considered issues around temporal availability, making explicit note of whether the data underpinning each variable was a single or repeat measure, and if repeat, recording the frequency with which the variable is updated (relevant column header: *Temporal availability)*.

We used our categorization to consider what types of environmental management problems our dataset is equipped to inform. We specifically looked for data gaps, noting the extent to which there was sufficient information across all parts of the system (social/human, natural/ecological and interactions between) to adequately ‘describe’ regions and to use techniques such as clustering to identify ‘similar’ regions. We conclude that the extent and condition of natural systems is well described, in particular for vegetation but less so for species. Human systems are also well described, although most variables describe individuals or households. There are no variables describing businesses or other organizations. There are only two variables describing environmental investments. The dataset does not contain variables that describe the outcomes of environmental interventions or the behaviors, objectives and values that drive environmental behaviors. Variables that describe how the social and ecological systems interact are relatively few.

Data gaps mean that our database cannot be used to address all environmental problems, but it is nevertheless clear that the breadth of available variables are more than sufficient to adequately describe and characterize social and ecological systems and interactions between systems, making this dataset a useful resource for Australian Natural Resource Managers. Our metadata file is thus a useful resource to any manager seeking national scale data that covers the range of variables they wish to use to describe their planning and management context and support spatial decision making.

Once satisfied that our dataset was sufficiently broad to adequately describe systems across the continent, we compiled all data into harmonized geospatial databases. While all datasets that we included had to meet our inclusion criteria of being a comprehensive national scale spatial dataset, each was collected in different ways at different scales, and often for different socio-political units or boundaries. We redressed the problem that arises when different agencies collect data at different geographic scales by writing bespoke algorithms within a GIS to ‘convert’ data that had been collected at one geographic scale, into indicators that could be used at other scales – ensuring consistent measures across the continent. For each dataset we chose the appropriate summary statistic – mean, max, min, or proportion of area, and calculated the statistic typically as a weighted average (mean, max, min) or proportion. Our metadata indicates the choice of summary statistic for each variable in the *Data summarized by* field.

We compiled all calculated indicators into two integrated datasets that contain 425 variables; one dataset describes 63 Natural Resource Management Regions (noting that only 56 of the 63 have complete data given many are offshore island areas); the other describes 419 (sub) bioregions (formally, IBRA – Interim Biogeographic Regionalisation for Australia) – noting also, that (as for NRMs) 10 of the IBRA regions are offshore areas with very little data available.

**Fig. 3:**
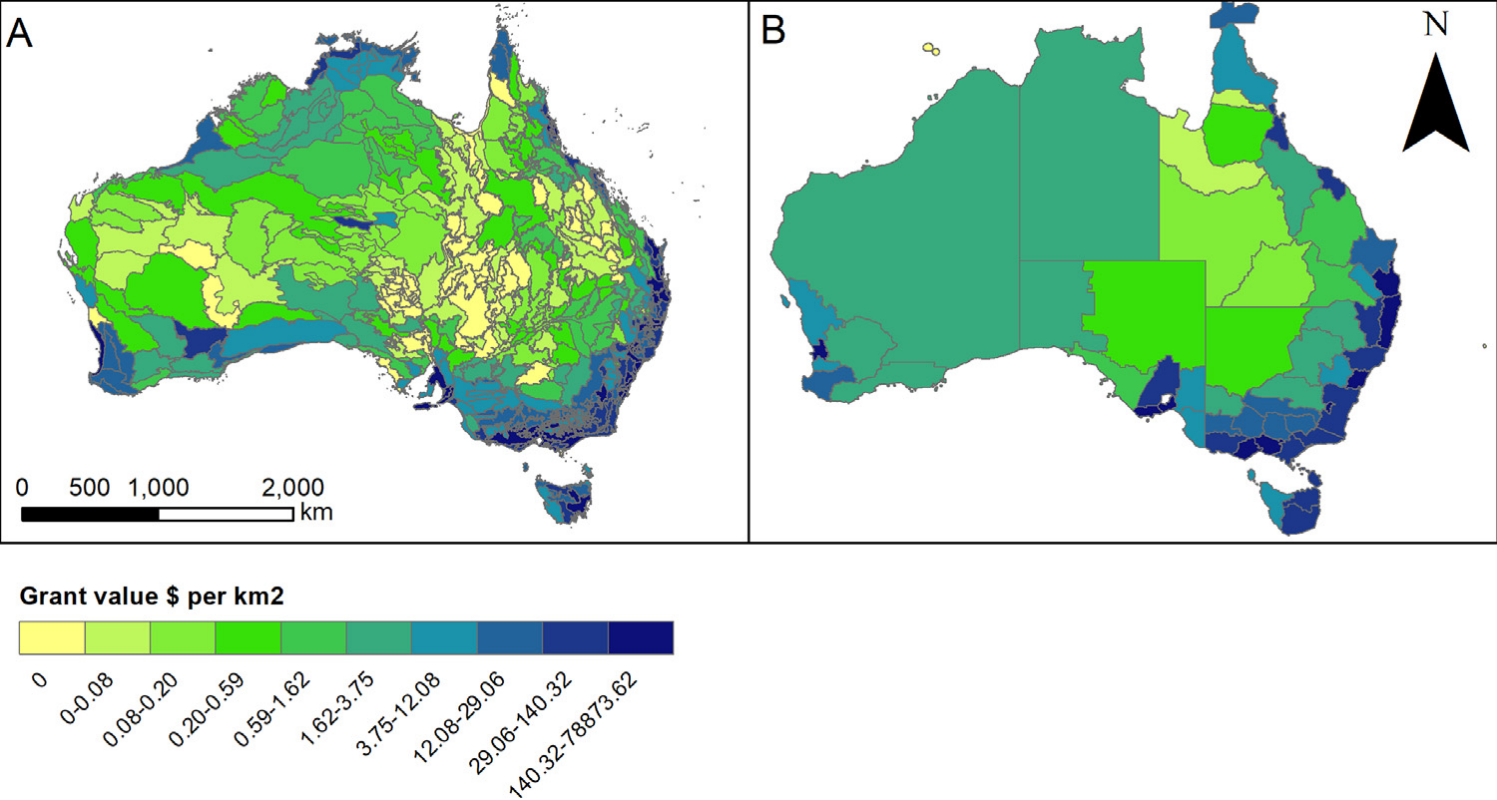
An example of visualization possible with supplied lpk files to support regional natural resource management. A) grant value ($ per km^2^) for IBRA subregions. B) grant value ($ per km^2^) for NRM regions.

At a national scale our compiled spatial data (provided in.lpk format to assist in visualization) provide a useful spatial interpretation of patterns of variability of indicators. For example, funds allocated to research, planning and on-ground environmental action must be spent cost-effectively to achieve the best outcomes for ecosystems and biodiversity and our dataset provides critically important contextual information to help target environmental investments (Fig. 3). Spatial patterns and inferences made diverge depending on scale of decision making and choices in visualization. For example, the grant values ($ per km2) at NRM region show moderate per km2 spending across the Northern Territory ($1.62 - $3.75 per km2; Fig. 3B) but when visualized at IBRA subregion it is evident that this spending is spatially heterogenous with much higher spending per km2 in coastal regions and inland around key natural values (Fig. 3A).

Our datasets are most suited to the task of supporting managers who make decisions on a relatively large geographic scale. They allow regions to be described using consistent and comparable metrics, so can be used to identify regions that may need specialized on-ground interventions (e.g., restoration, conservation, particular types of planning to negotiate competing interests). Our datasets can also be used to identify regions that are well suited to particular types of planning approaches and/or that share similar social and/or ecological characteristics, where knowledge sharing opportunities (including, transferring research findings from one place to another) are particularly promising – see [39], [40], [41], [42], [43], [44] for applied examples.

## Limitations

Data deficiencies prevent us from being able to report on all variables for all regions, in particular offshore island areas and smaller geographic IBRA regions. Missing data in these regions was mostly household variables where for some regions the number of households is so small that agencies either do not collect or do not report data at household level for privacy reasons. We have chosen to collate the data for two time steps (2016, 2021). Users should consider the time step most relevant to their analyses when selecting which variables to use in the data.

## Ethics Statements

The current work meets the ethical requirements for publication in Data in Brief and does not involve human subjects, animal experiments, or any data collected from social media platforms.

## CRediT authorship contribution statement

**Vanessa M. Adams:** Conceptualization, Methodology, Formal analysis, Writing – original draft, Writing – review & editing, Visualization, Funding acquisition. **Stuart Allen:** Methodology, Validation, Formal analysis, Data curation, Writing – review & editing. **Ruth Steel:** Investigation, Data curation, Writing – review & editing. **Natalie Stoeckl:** Conceptualization, Methodology, Investigation, Data curation, Writing – original draft, Writing – review & editing, Funding acquisition. **Silva Larson:** Conceptualization, Methodology, Investigation, Data curation, Writing – original draft, Writing – review & editing.

## Data Availability

Integrated data for natural resource managers - V.2.0 (Original data) (UTAS Research Data Portal)
